# Microstructure Evolution and Fretting Wear Mechanisms of Steels Undergoing Oscillatory Sliding Contact in Dry Atmosphere

**DOI:** 10.3390/ma17081737

**Published:** 2024-04-10

**Authors:** Alyssa A. Maich, Ronald Gronsky, Kyriakos Komvopoulos

**Affiliations:** 1 Department of Materials Science and Engineering, University of California, Berkeley, CA 94720, USA; 2 Department of Mechanical Engineering, University of California, Berkeley, CA 94720, USA

**Keywords:** cracks, dislocation cell walls, fretting, microstructure, oxidation, steel, phase transformation, wear mechanisms

## Abstract

Variations in the microstructure and the dominant fretting wear mechanisms of carbon steel alloy in oscillatory sliding contact against stainless steel in a dry atmosphere were evaluated by various mechanical testing and microanalytical methods. These included scanning electron microscopy and energy dispersive spectrometry with corresponding elemental maps of the wear tracks, in conjunction with cross-sectional transmission electron microscopy of samples prepared by focused ion beam machining to assess subsurface and through-thickness changes in microstructure, all as a function of applied load and sliding time. Heavily dislocated layered microstructures were observed below the wear tracks to vary with both the load and sliding time. During the accumulation of fretting cycles, the subsurface microstructure evolved into stable dislocation cells with cell walls aligned parallel to the surface and the sliding direction. The thickness of the damaged subsurface region increased with the load, consistent with the depth distribution of the maximum shear stress. The primary surface oxide evolved as Fe_2_O_3_ and Fe_3_O_4_ with increasing sliding time, leading to the formation of a uniform oxide scale at the sliding surface. It is possible that the development of the dislocation cell structure in the subsurface also enhanced oxidation by pipe diffusion along dislocation cores. The results of this study reveal complex phase changes affecting the wear resistance of steels undergoing fretting wear, which involve a synergy between oxidative wear, crack initiation, and crack growth along dislocation cell walls due to the high strains accumulating under high loads and/or prolonged surface sliding.

## 1. Introduction

Wear may occur by one or more mechanisms depending on various factors, such as applied load, elastic-plastic material properties, and environmental conditions. The most commonly encountered wear mechanisms are abrasion, adhesion, tribochemical (oxidational or corrosive) reactions, contact fatigue, and delamination [1,2,3]. Most wear processes usually involve a combination of the foregoing wear mechanisms, with one often dominating the steady-state wear behavior. Microscopy may be used to visually distinguish the dominant wear mechanism(s), including changes in the subsurface microstructure caused by wear processes. For example, microstructural changes may significantly alter the hardness of the material, and if adhesion or abrasion is the prevailing wear mechanism, a change in hardness can greatly affect the wear rate [1,2]. In the case of delamination wear, a change in microstructure, including, but not limited to, the rearrangement and creation of dislocations, can have a profound effect on the development of plasticity and, ultimately, on the evolution of subsurface cracking [4]. For systems where tribochemical reactions are prevalent, a change in microstructure can influence strain-dependent oxidative properties, generating pathways for oxidation to occur more destructively [5,6]. Accordingly, since the wear process is affected by microstructural changes, identifying the dominant wear mechanism(s) requires detailed characterization of the subsurface microstructure, which is controlled by the stresses and strains generated during sliding.

Stress-activated microstructural transformations exhibit similarities with those instigated by temperature changes. Interstitial atoms like carbon and nitrogen can diffuse under the large strain gradients generated by the stress fields of dislocations, impeding further dislocation motion. Minimum-energy dislocation configurations include sub-boundaries surrounded by high concentrations of interstitial atoms, leading to the formation of phases with compositions different from that of the matrix, such as carbide precipitation in martensite. Severe plastic deformation may cause carbides to dissociate back into the matrix, either by decomposition based on the Gibbs–Thompson effect [7] or through carbon-dislocation interactions [8,9], making knowledge of microstructural evolution under specific application conditions critical to the longevity of engineering components.

Despite several studies of surface damage instigated by wear, knowledge of subsurface features, such as oxidation depth, microstructural modification, extent of plasticity, and subsurface wear mechanisms is relatively sparse. This could be attributed to the difficulty of extracting cross-sectional samples without introducing damage artifacts and the underutilization of high-resolution microanalytical techniques. The situation becomes even more challenging in the case of material loss under small-amplitude oscillatory contact conditions, known as fretting wear [10,11]. This type of wear is responsible for catastrophic failure in numerous applications, including industrial machinery, turbine engine parts, microelectromechanical devices, circuit boards, micro-relays, micro-switches, and other contact-mode components where a source of vibration is present [12,13,14]. Fretting wear can also increase the clearance between adjacent mechanical elements in applications where tight tolerances are critical to effective operation, or cause jamming when wear debris is trapped at the contact interface, as in the instance of engine rotating assemblies and journal bearings [15]. Plastic strains produced during fretting induce near-surface damage, which can be significantly different from that in the bulk. Under fretting conditions, fatigue cracks can grow from the surface into the bulk of the material, or in the subsurface parallel to the sliding direction for a significant time during fatigue loading before they eventually veer off to the surface and/or intersect with other cracks to cause delamination. Fretting wear may also have an environmental component, influenced by several factors, such as temperature, humidity, and others [16,17,18]. In spite of several fretting wear studies, the mechanisms controlling fretting wear are still not well understood. In most investigations of fretting wear, the focus was on the damaged surface; however, subsurface damage must be considered in conjunction with surface damage to elucidate the mechanisms of this type of wear process. Specifically, very little is known about changes in the subsurface microstructure of steels caused by fretting wear.

Microstructural modification can affect the response of a material to plastic deformation, consequently influencing the oxidation rate. Changes in oxidation rate may, in sequence, alter the wear behavior. The role of oxidation during fretting wear and damage accumulation remains a controversial subject [19,20,21,22,23]. Fretting wear has been thought to mainly be an oxidative process; however, it has been discovered that fretting wear can also occur in noble metals [24]. The significant increase of the wear rate with the slip amplitude encountered under fretting wear conditions does not appear to coincide with an oxidative wear process, which is typically perceived as a mild wear process, because the oxide scale can act as a lubricious (low adhesion) solid film [25,26,27] that decelerates the wear process. Despite numerous oxidational wear models developed for frictional heating, oxidational wear stimulated by mechanical stress/strain effects generated by surface tractions in fretting wear is much less understood than thermally induced oxidational wear.

The objective of this study was to bridge the knowledge gap between surface and subsurface wear mechanisms and stress-induced microstructural alterations in steel undergoing fretting wear. This was accomplished through detailed examination of surface wear features using scanning electron microscopy (SEM) and cross-sectional samples examined with focused ion beam/transmission electron microscopy (FIB/TEM) and energy dispersive spectrometry (EDS). Subsurface microstructural changes and fretting wear mechanisms are correlated with the applied load and sliding time. TEM micrographs provide insight into the extent of damage below the sliding interface, microstructure modification depth, and wear features intrinsic to particular wear mechanisms. A multilayered steel structure extending to a depth controlled by the surface tractions and exhibiting multiple phase transformations is observed to evolve with the progression of fretting wear. Elemental EDS maps illuminate the evolution of oxidational wear and material transfer between the sliding steel surfaces. The FIB/TEM results are interpreted in the context of multiple stress-induced phase changes influencing the fretting wear resistance of sliding steel surfaces.

## 2. Materials and Experimental Methods

### 2.1. Specimens

Motivated by the problems of fretting wear plaguing the gas turbine industry, samples of two alloys that make contact during turbine operation, namely A216 steel (rotor-blade rings) and 410 stainless steel (diaphragm couplings), with compositions given in Table 1, were chosen for the present study. Disks of A216 steel with a diameter equal to 2 cm and pins of 410 stainless steel with a diameter equal to ~1 cm were polished with progressively finer abrasive papers (240–1200 grit size), followed by 6 and 1 μm diamond paste. While chromium is merely one of the trace elements in the A216 carbon steel disk, its high content in the 410 stainless steel provided a method for analyzing transfer of the pin material to the disk surface using spatially resolved EDS.

### 2.2. Mechanical Testing

Fretting wear experiments were performed with a pin-on-disk testing apparatus (UMT3, Bruker, Goleta, CA, USA). The input parameters were the load (2, 10, and 15 N), oscillation frequency (5 Hz), oscillation amplitude (120 µm), and test time (1–69 h), whereas the measured parameters were the friction force and the lateral displacement. Preliminary testing for smaller oscillation amplitudes revealed a linear variation of fretting wear rate with oscillation amplitude. Since fretting wear is typically encountered for oscillation amplitudes of less than 200–300 μm, the oscillation amplitude in the wear experiments was fixed at 120 μm to simulate fretting wear conditions characterized by both slip and stick-slip behaviors in the 2–15 N load range. 

From an analysis of the friction force versus lateral distance hysteresis curves, the responses for 2 and 10 N load were found to be in slip mode, whereas those for 15 N load were found to be in stick-slip mode. Since the friction force versus reciprocating distance hysteresis remained stable after sliding for 12 h, a sliding time of ≥12 h will be hereafter associated with steady-state fretting wear, whereas the responses for a sliding time much less than 12 h will be attributed to so-called transient fretting wear.

### 2.3. Surface Profilometry

Three-dimensional images of the wear tracks were obtained with a mechanical stylus surface profilometer (P-10, KLA-Tencor, Milpitas, CA, USA) having a conical diamond tip with a radius of curvature equal to 2 μm. In all surface scans, a light load of 50 μN was applied to the diamond tip to preserve the integrity of the sample surfaces.

### 2.4. Microanalytical Methods

Studies by SEM and EDS were performed in a scanning electron microscope (EVO MA-10, Zeiss, Oberkochen, Germany) with a tungsten filament operated at 20 kV. The SEM was used to identify the dominant wear mechanisms, illuminated by characteristic wear track features. X-ray analysis was conducted with an EDAX system (Genesis, Mahwah, NJ, USA) having a silicon drift detector. This method was used to study surface oxidation associated with fretting and material transfer between the sliding surfaces through mapping of chromium.

**Figure 1:**
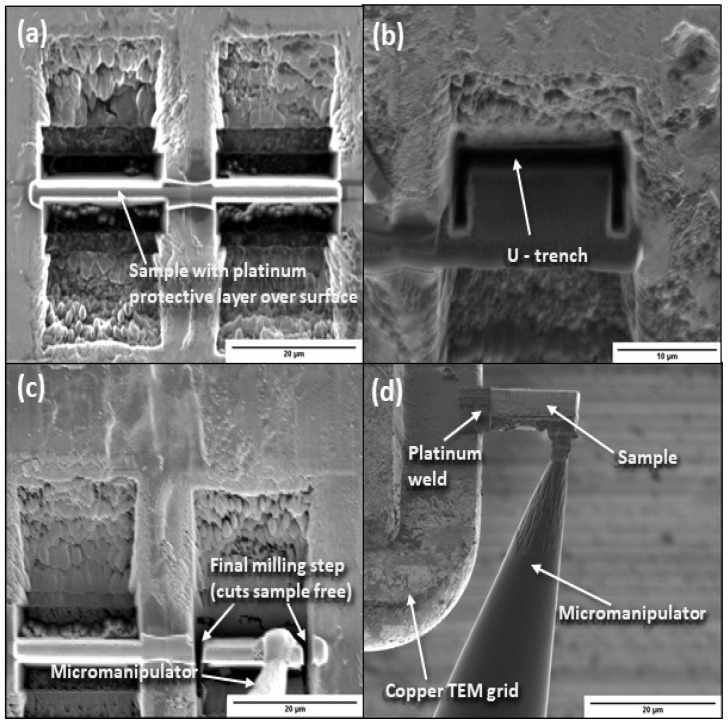
Cross-sectioning process using a focused ion beam: (**a**) sample milling to a thickness of less than 500 nm and length equal to about 20 μm, (**b**) cutting of a u-trench below the tilted sample, (**c**) micromanipulator welding to the platinum layer deposited onto the sample surface and milling of the remaining sample, and (**d**) sample removal from the trench, thinning down to electron transparency, and welding to a copper TEM grid.

For subsurface damage analysis, cross-sectional samples were prepared with a 3D FIB/TEM system (Quanta, FEI, Hillsboro, OR, USA) using a gun voltage of 30 kV. Cross-sectional samples were obtained parallel and perpendicular to the wear track direction. Figure 1 illustrates the FIB milling process used to create the TEM samples. First, a protective platinum layer was deposited onto the sample surface by Ga^+^ ion deposition (current density = 2 pA/μm^2^) and a rough trench was milled, using a current of 30 nA (Figure 1a). The current gradually decreased to 0.5 nA as the beam distance from the sample surface was reduced to minimize damage of the sample before extraction. When the thickness was reduced to ~500 nm, the sample was removed, and a U-trench was cut below the tilted sample (Figure 1b). A micromanipulator was then welded to the platinum layer, and the remaining sample was milled free (Figure 1c). Subsequently, the sample was carefully maneuvered out of the trench with the micromanipulator and welded to a copper TEM grid using a platinum layer (Figure 1d). The micromanipulator was then cut, and the sample was thinned down to electron transparency to enable the analysis in the electron microscope. 

The subsurface microstructure and wear features were examined with a TEM (2011 LaB6, JEOL, Peabody, MA, USA) operated at 200 kV. The TEM was also used to determine the thickness of the oxide scale and the damage depth and to examine the dislocation substructure and distribution in the sample.

## 3. Results

Experimental results of this investigation are grouped into three main subsections centered on the evolution of the friction force and friction hysteresis, the surface and subsurface wear mechanisms, the development of plasticity, and the microstructural changes caused by the oscillatory sliding process under different loads.

### 3.1. Friction

**Figure 2:**
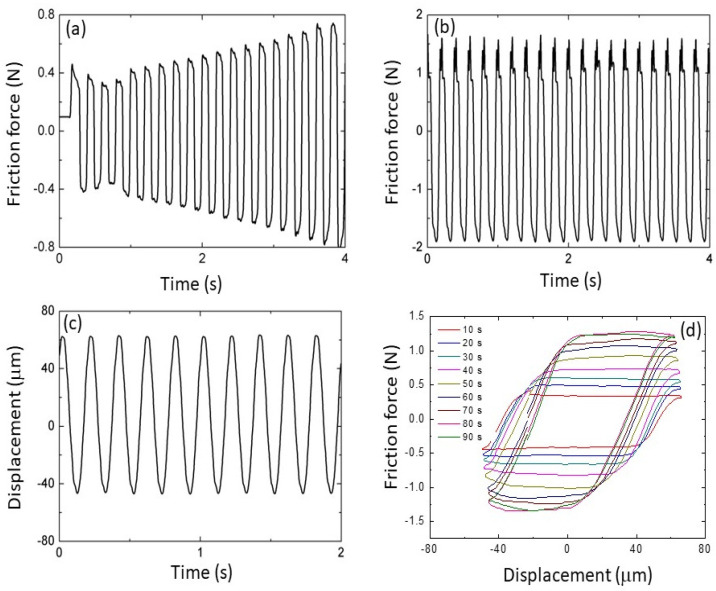
Variation of the friction force during (**a**) the initial stage of sliding and (**b**) at steady-state sliding. (**c**) Oscillatory lateral displacement versus time. (**d**) Evolution of friction force-lateral displacement hysteresis during the initial stage of oscillatory sliding.

The friction force demonstrated a rapid increase from the instigation of oscillatory sliding (Figure 2a), reaching a steady state within a few minutes of testing (Figure 2b) under conditions of fixed load and controlled lateral displacement of the pin sample (Figure 2c). The variation of the friction force with the lateral displacement displayed a hysteresis typical of oscillatory sliding contact that rapidly grew in size with time (Figure 2d), indicating the fast accumulation of plastic deformation. The intensification of the friction force, exemplified by the results shown in Figure 2, indicates the progression of external energy dissipation in the steel samples with accumulating oscillation cycles.

### 3.2. Wear

**Figure 3:**
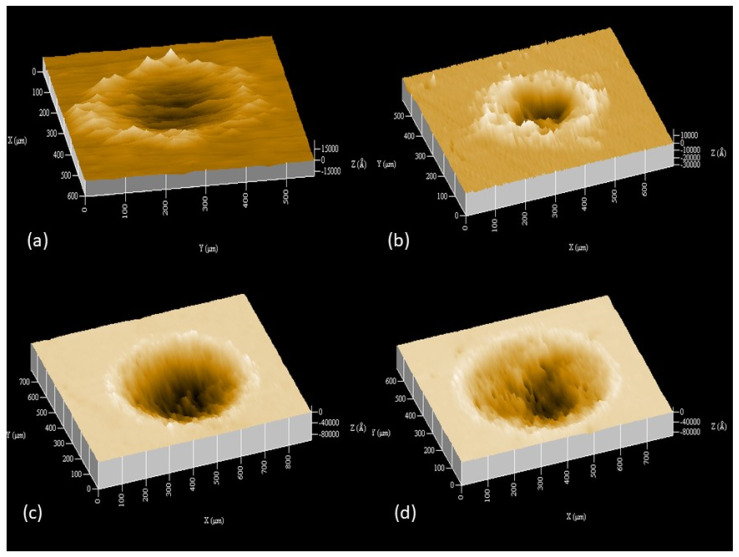
Three-dimensional images of wear tracks on A216 carbon steel disks obtained under steady-state oscillatory sliding for a load equal to (**a**) 0.5 N, (**b**) 2 N, (**c**) 10 N, and (**d**) 15 N.

**Figure 4:**
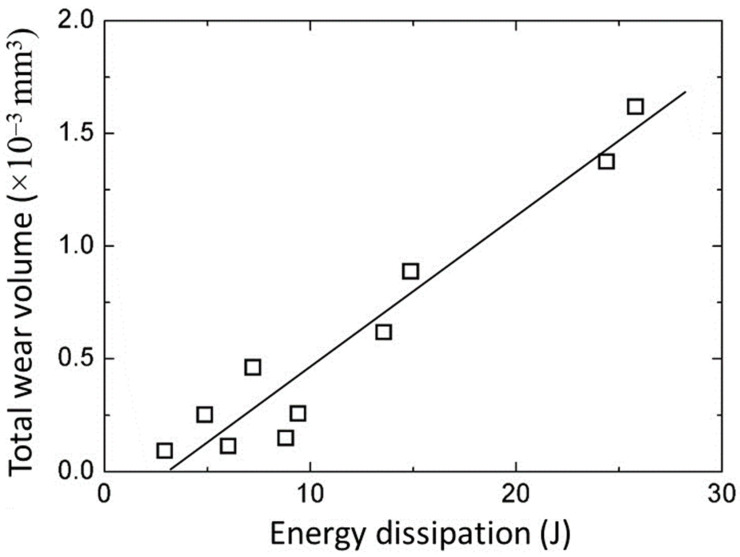
Total wear volume versus energy dissipation under steady-state oscillatory sliding conditions.

Figure 3 shows representative images of wear tracks on steel disks produced after sliding for 12 h. As expected, a deeper wear scar formed under a higher load. Figure 4 shows a monotonic increase of the total wear volume with energy dissipation during steady-state oscillatory sliding. The total wear volume represents the material loss from both the disk and the pin samples, computed from 3D surface profilometry images (e.g., Figure 3) and spherical cap volume calculations based on the known pin radius and the measured diameter of the circular wear scar, respectively. The energy dissipation was computed as the area of the force-displacement hysteresis at steady-state sliding under a given load. The trend for the loss of material to increase with energy dissipation is consistent with the increase of the wear volume with the applied load and the sliding distance (time) or number of oscillation cycles.

### 3.3. Surface Wear Characteristics

**Figure 5:**
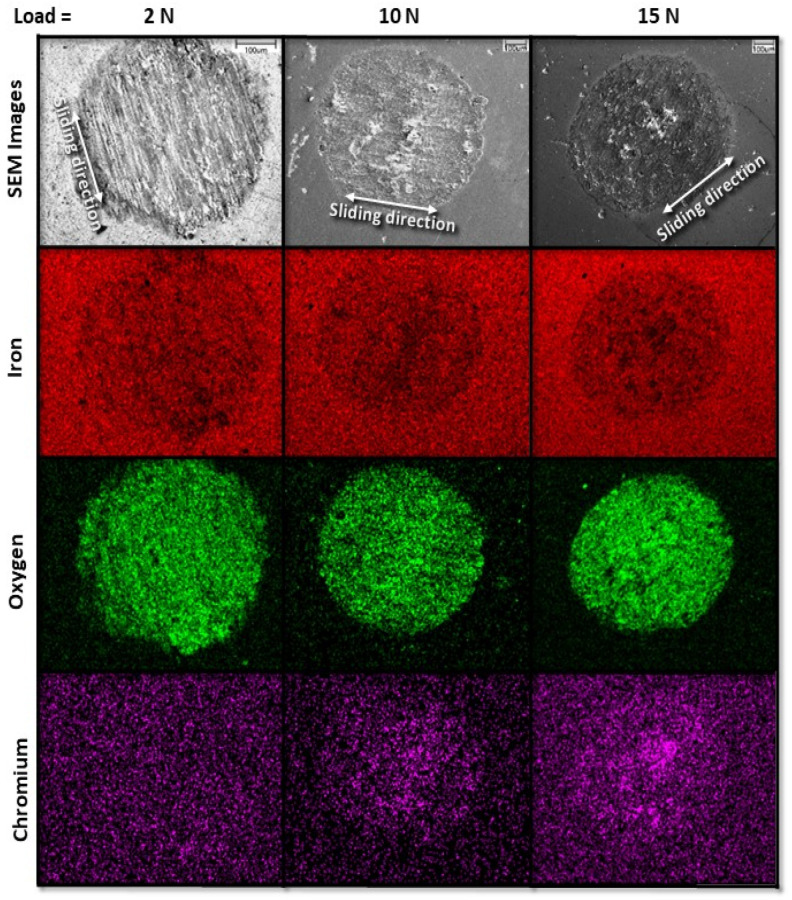
SEM images of the entire wear scar produced on the surface of A216 carbon steel disk (first row) and EDS elemental maps (second, third, and fourth rows) for 2, 10, and 15 N load and 12 h sliding time.

Figure 5 qualitatively shows the dependence of fretting wear on load after sliding for 12 h. The first row displays SEM micrographs of wear scars produced as a result of oscillatory sliding under a load of 2, 10, and 15 N, whereas the second, third, and fourth rows encompass corresponding EDS maps of iron, oxygen, and chromium, respectively. The SEM images and EDS maps reveal an enhancement of oxidation and material transfer from the pin specimen (illustrated by the chromium maps) onto the disk wear scars with increasing load. The oxygen maps indicate that although the oxidation of the sliding steel surface exhibits similarities for a load of 2 and 10 N (slip), oxidation was more pronounced for a load of 15 N (stick-slip), suggesting that the transition from slip to stick-slip conditions due to the load increase was conducive to oxidation. Material transfer from the pin to the disk surface (tracked by EDS) also increased with load, regardless of slip (2 or 10 N load) or stick-slip (15 N load) contact conditions. The high-magnification SEM images and EDS maps demonstrate a change in dominant wear mechanisms from adhesive and abrasive wear at relatively low (2 N) or intermediate (10 N) loads to abrasive wear and material transfer from the pin due to adhesive wear in the slip region of the disk due to sliding under a high load (15 N). The oxygen and chromium EDS maps demonstrate that the load increase promoted surface oxidation and material transfer, respectively.

**Figure 6:**
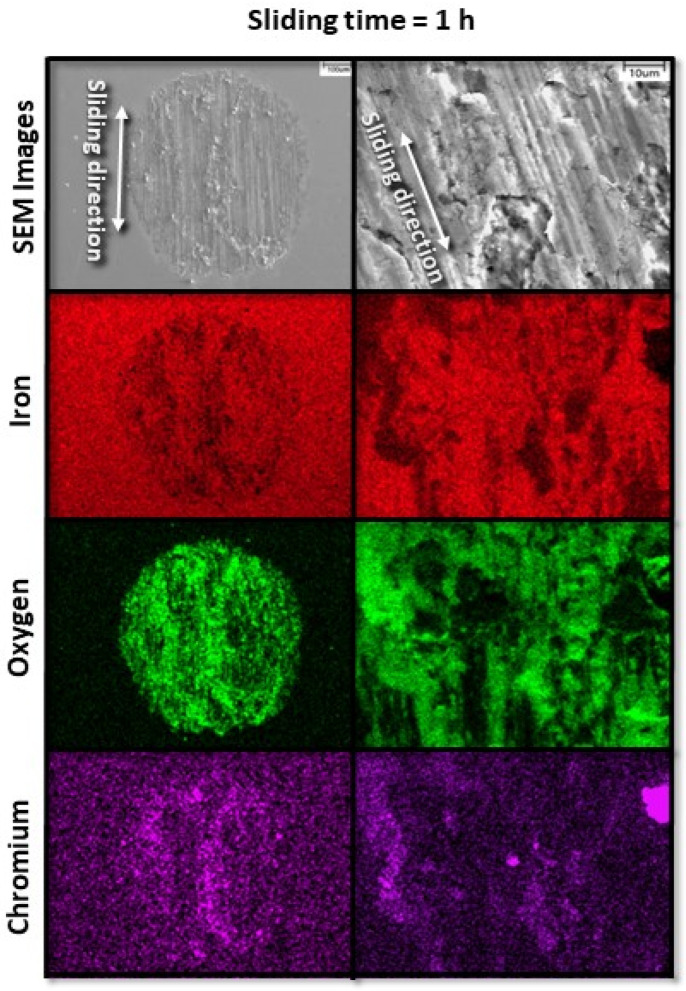
Low- and high-magnification SEM images (first row) and corresponding EDS elemental maps (second, third, and fourth rows) revealing characteristic wear features on the surface of A216 carbon steel disk for 10 N load and 1 h sliding time.

**Figure 7:**
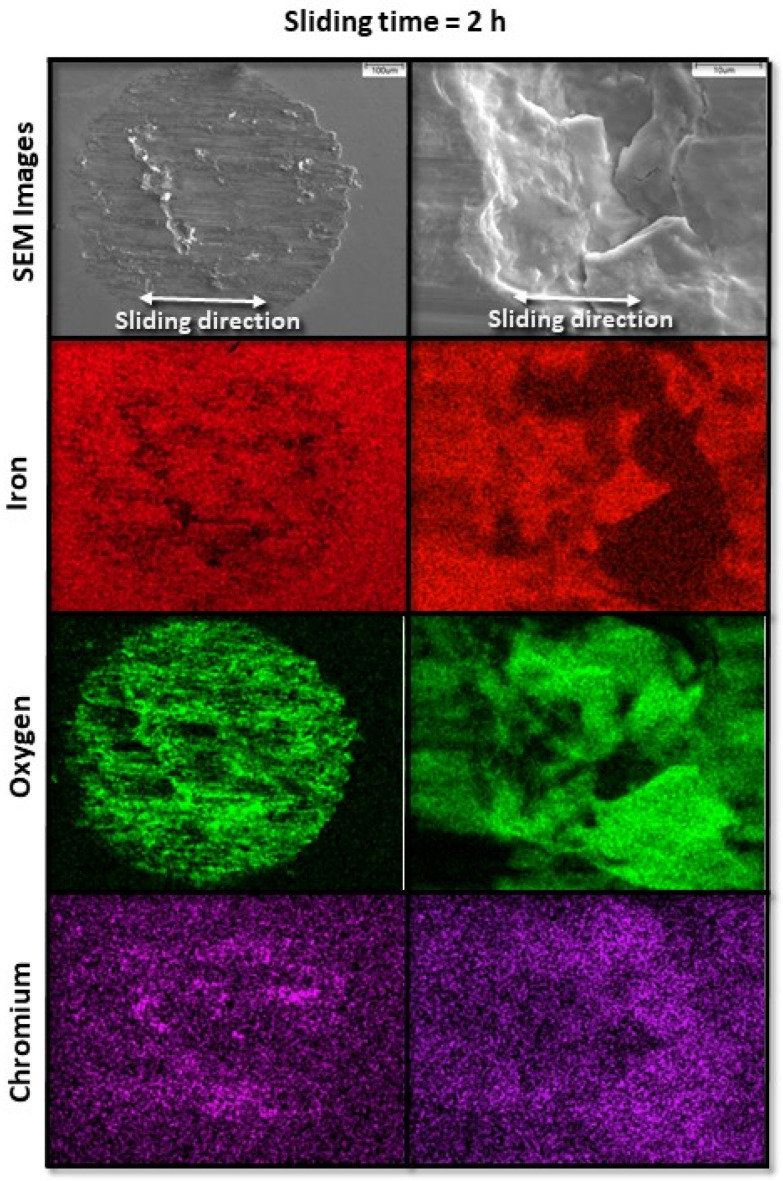
Low- and high-magnification SEM images (first row) and corresponding EDS elemental maps (second, third, and fourth rows) revealing characteristic wear features on the surface of A216 carbon steel disk for 10 N load and 2 h sliding time.

Figure 6 and Figure 7 show the evolution of fretting wear and oxide formation for a 10 N load (pure slip) and sliding time equal to 1 and 2 h, respectively. The chromium maps confirm the transfer of material from the pin to the disk surface, where it was oxidized along with iron. Sliding for 1 and 2 h corresponded to the transient (run-in) stage of fretting wear, characterized by a progressively increasing conformity of the wearing surfaces and localized high-pressure spikes, as suggested by the wear features found on corresponding sliding tracks. 

**Figure 8:**
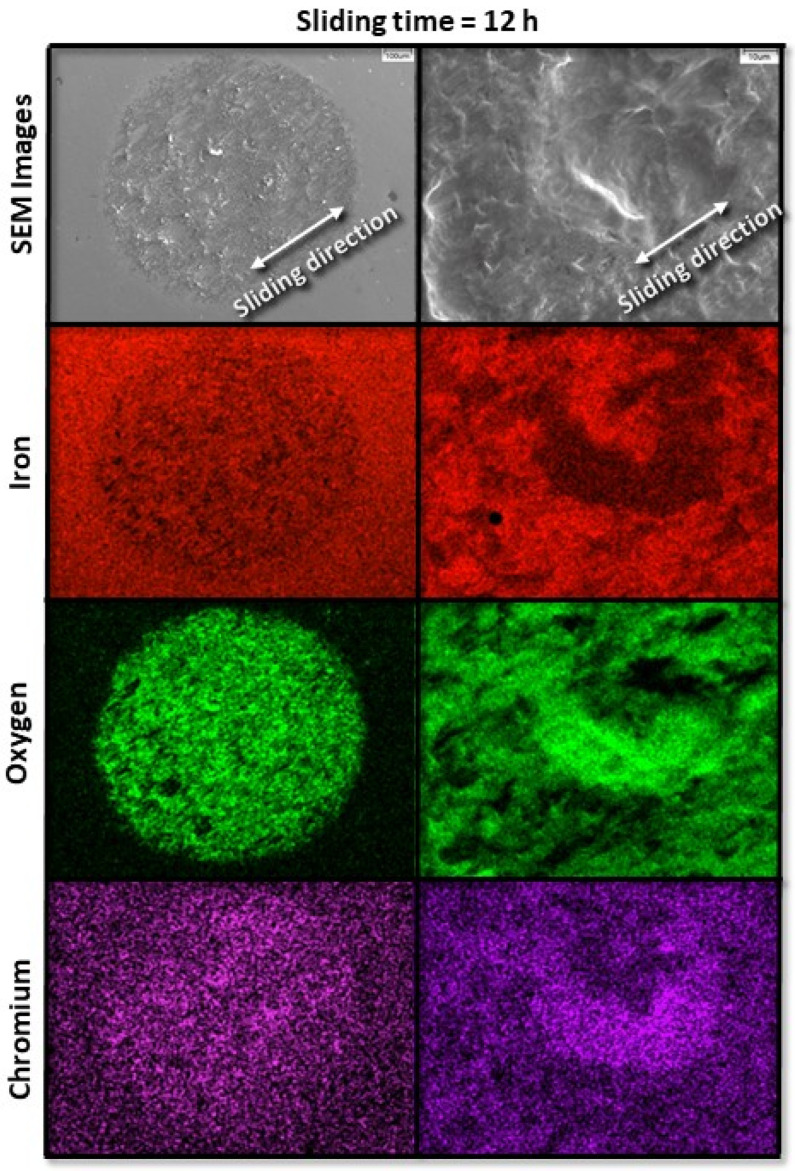
Low- and high-magnification SEM images (first row) and corresponding EDS elemental maps (second, third, and fourth rows) revealing characteristic wear features on the surface of the A216 carbon steel disk for 10 N load and 12 h sliding time.

**Figure 9:**
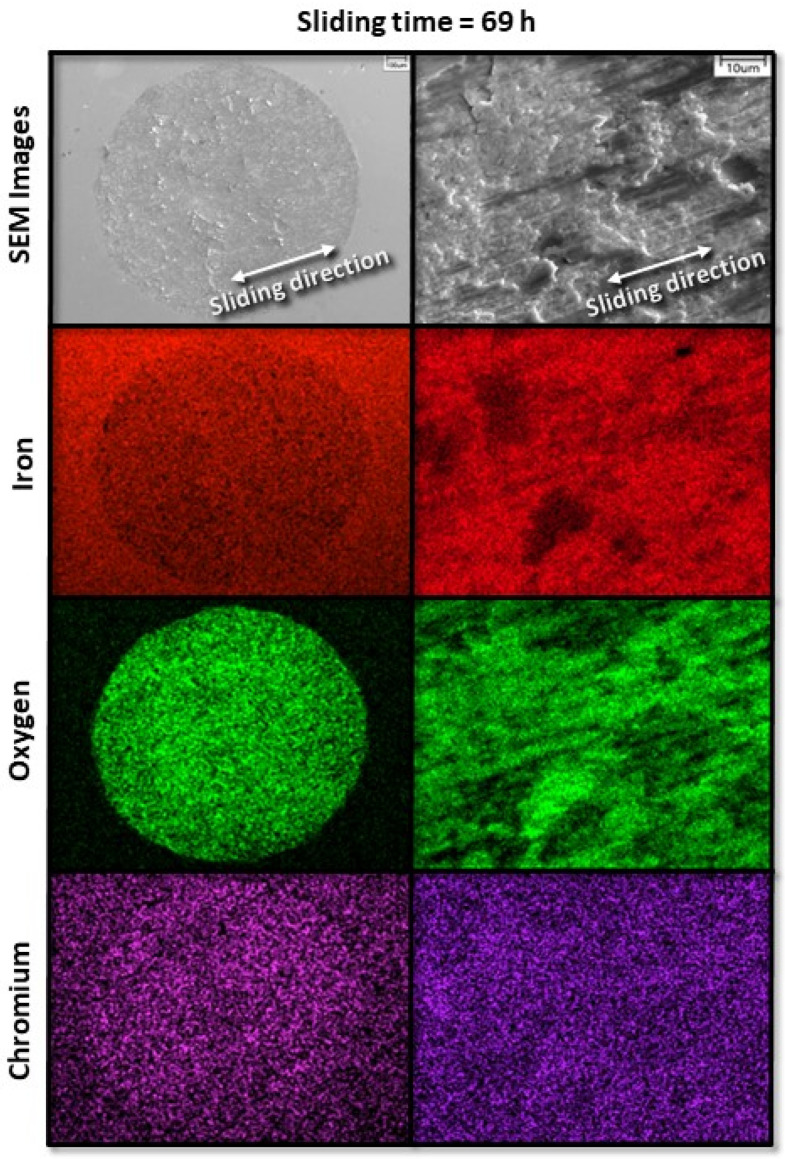
Low- and high-magnification SEM images (first row) and corresponding EDS elemental maps (second, third, and fourth rows) revealing characteristic wear features on the surface of A216 carbon steel disk for 10 N load and 69 h sliding time.

Figure 8 and Figure 9 show the progression of fretting wear and oxide formation for a 10 N load (pure slip) after 12 and 69 h of sliding, respectively. The longer duration of sliding resulted in more uniform oxidation with relatively less chromium transfer from the pin to the disk surface. However, after sliding for 12 h, there are still some areas of heavier chromium transfer. After 69 h of sliding (steady state), the surface exhibits a more uniform appearance in both oxidation and chromium transfer, with very little chromium transfer from the pin to the disk and the formation of a uniform oxide layer on the disk surface.

### 3.4. Subsurface Wear Characteristics

**Figure 10:**
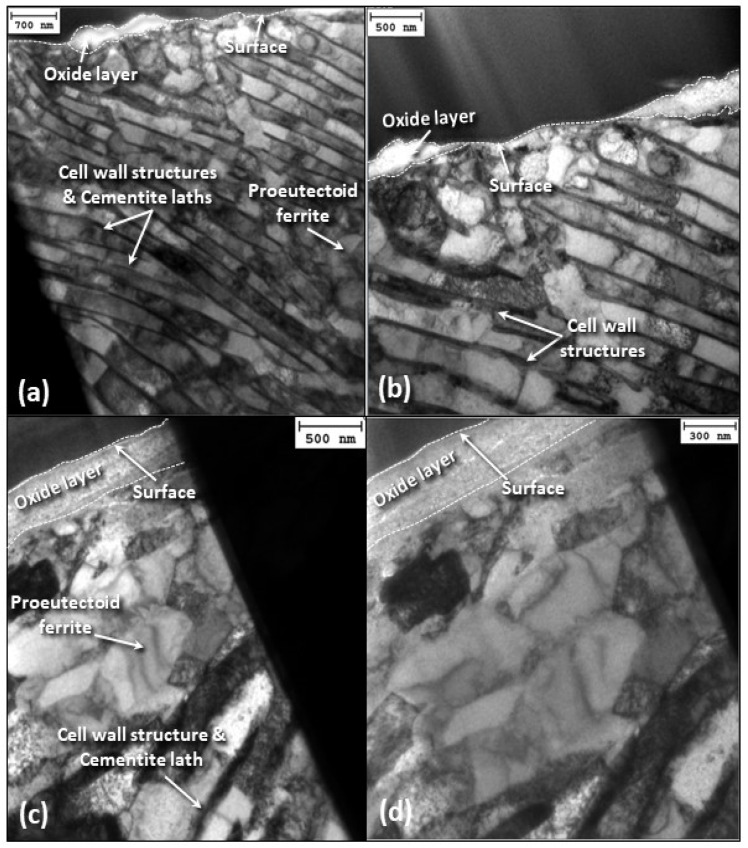
Cross-sectional bright-field TEM images of the subsurface microstructure of A216 carbon steel disk oriented (**a**,**b**) parallel and (**c**,**d**) perpendicular to the oscillation direction for 15 N load and 12 h sliding time.

Figure 10 shows bright-field TEM images of cross sections obtained parallel and perpendicular to the oscillation direction for 15 N load and 12 h sliding time. The images display a hypoeutectoid microstructure containing proeutectoid ferrite and pearlite, which is the expected microstructure for carbon steel, with dislocation cells running through the two phases. A cell wall structure is also evinced below the sliding surface. The most significant microstructural changes in the direction parallel to the oscillation direction appear to be at an angle of ~45° from the surface. Uneven surface coverage by the oxide scale is also observable at the sliding surface.

**Figure 11:**
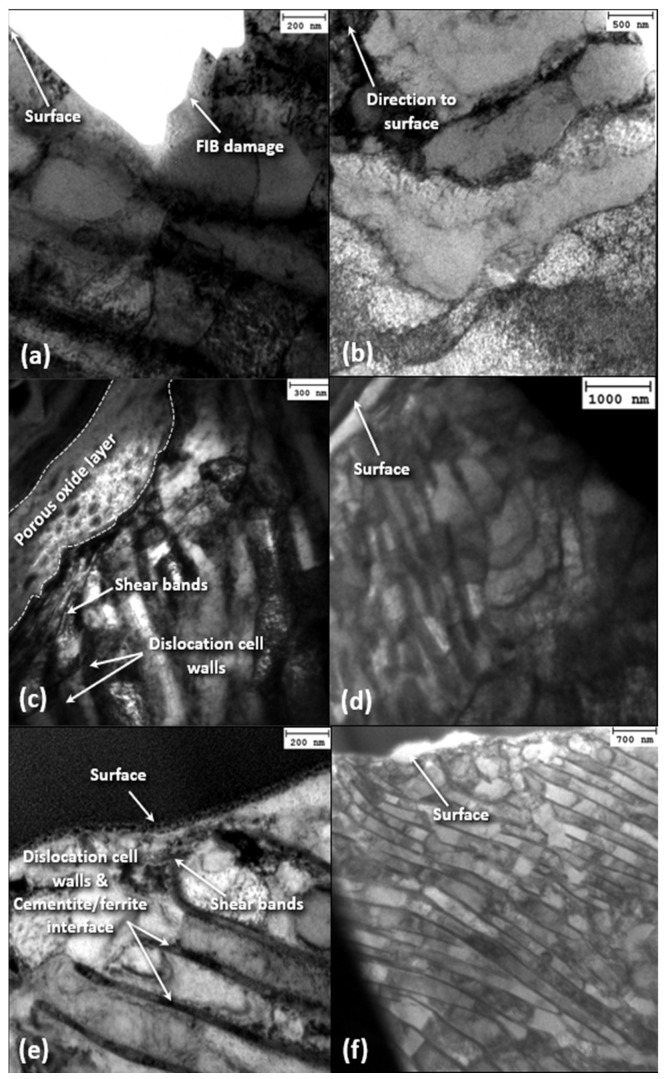
Bright-field TEM images of (**a**,**b**) original and (**c**–**e**) deformed near-surface (left) and mid cross-sectional (right) microstructures of A216 carbon steel disk samples parallel to the oscillation direction for 12 h sliding time and (**c**,**d**) 2 N and (**e**,**f**) 15 N load.

Figure 11 shows bright-field TEM images of cross sections of original and deformed carbon steel microstructures obtained parallel and perpendicular to the oscillation direction for 2 and 15 N load and 12 h sliding time. The undeformed microstructure in the depth range of 1.5–3 μm is that of pearlitic steel. Oscillatory sliding under a load of 2 N augmented the density of dislocation cell walls compared to the undeformed microstructure, with the dislocation wall angle remaining at ~45°. Increasing the load to 15 N produced dense dislocation walls with fewer dislocations forming between the walls. The oxide scale seems to be quite porous in both specimens tested under a load of 2 and 15 N, with shear bands below the oxide contributing to its rupture from the sliding surface.

**Figure 12:**
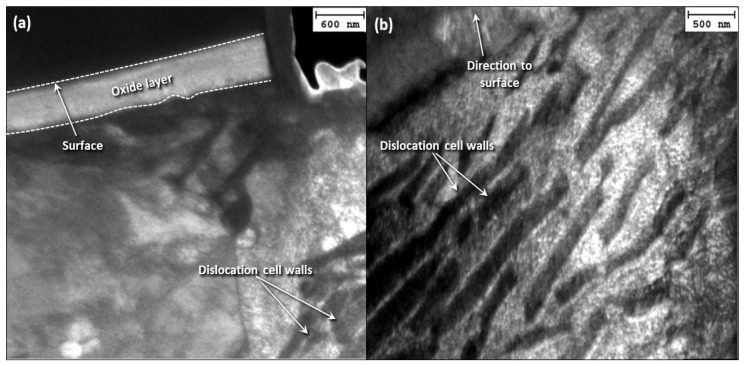
Dark-field TEM images of the deformed subsurface microstructure of A216 carbon steel disk in (**a**) near-surface and (**b**) mid-section cross sections parallel to the oscillation direction for 2 N load and 69 h sliding time.

Figure 12 shows dislocation cell configurations in near-surface and mid-section planes parallel to the oscillation direction for 69 h (steady state) sliding time and 2 N load. The microstructure produced after sliding for 12 h under a 2 N load (Figure 11c) displays denser dislocation walls compared to the unworn microstructure (Figure 11a), with a dislocation band angle equal to ~45°. However, the steady-state microstructure demonstrates significantly increased dislocation density, more closely spaced dislocation walls that have rotated to a direction nearly parallel to the surface, and a fairly even dislocation density between the cell walls. The area without dislocation cell walls adjacent to the sliding surface of the steady-state microstructure may be attributed to a grain orientation resulting in fewer slip systems, which enhanced the deformation resistance. A pearlite structure is not detected in the areas of dislocation cell walls in the steady-state microstructure. A quite porous oxide scale exhibiting uneven surface coverage formed during the transient stage of fretting wear, which became denser and evenly covered the sliding surface at steady state, indicating the evolution of controllable oxidation wear. Another characteristic feature of the steady-state microstructure is the development of shear bands directly below the oxide scale.

**Figure 13:**
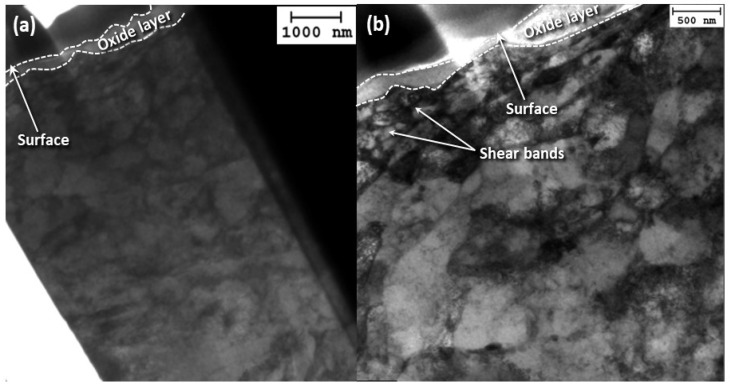
Bright-field TEM images of the deformed subsurface microstructure of 410 stainless-steel pin at cross sections parallel to the oscillation direction showing (**a**) the formation of an oxide layer and (**b**) the formation of shear bands close to the sliding surface for 2 N load and 12 h sliding time.

Figure 13 shows cross-sectional TEM images of the stainless-steel pin microstructure in sections parallel to the oscillation direction for 2 N load and 12 h sliding time. Contrary to the carbon steel disk, the images do not reveal the development of dislocation cell walls in the stainless-steel microstructure.

## 4. Discussion

Fretting wear is often described as an oxidational wear process, initially designated as fretting corrosion. Fretting wear shows an increase in material removal rate not seen in typical sliding wear processes, which has not been explained comprehensively. Since oxides may act as solid lubricants during sliding, oxidational wear may be considered as mild wear; however, this does not fit the conventional definition of fretting wear. The results of this study indicate a synergy between oxidational wear and subsurface fatigue. Nevertheless, the subsurface dislocation structure also played an important role in the wear process by enhancing the formation of different oxides with accumulating fretting cycles and the detachment of material through the development of dislocation cells. Therefore, to elucidate the fretting wear process, it is necessary to provide insight into the oxidation process and associated subsurface microstructural evolution. For that reason, an in-depth analysis of the role of dislocation cells, microstructural evolution, oxidative wear, and material transfer in fretting wear is presented below.

### 4.1. Dislocation Cells/Persistent Slip Bands

**Figure 14:**
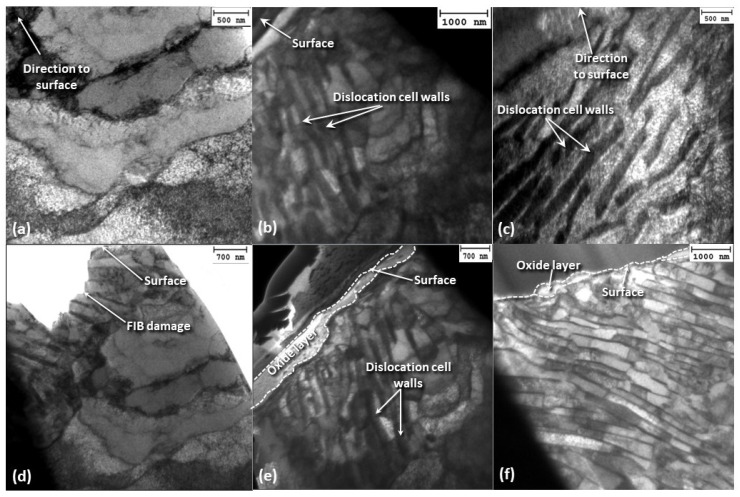
Bright-field TEM images illustrating the development of persistent slip bands in A216 carbon steel disk during fretting wear for 2 N load and sliding time equal to (**a**) 0, (**b**) 12 h, and (**c**) 69 h and for 12 h sliding time and load equal to (**d**) 0, (**e**) 2 N, and (**f**) 15 N.

Distinctive microstructure and subsurface features were observed in the fretting wear experiments of this study. Indeed, a wall-like (persistent slip band) structure emerged at varying depths with increasing sliding time (Figure 14a–c) and depending on the applied load (Figure 14d–f). These wall-like bands emerged during plastic deformation [28] and could be associated with the spatial variation of the dislocation density in the deformed subsurface. When the dislocations arrange themselves into a crystallographic planar wall configuration, they are most often found to persist in this morphology during subsequent plastic deformation. Dislocation walls of this type are, therefore, also known as persistent slip bands. Alternatively, dislocations may arrange themselves into arrays that do not align so sharply, but form meandering interfaces known as dislocation cells or subgrain boundaries, which affect the strain hardening behavior of metals [29]. The driving force for the formation of both persistent slip bands and dislocation cells is the reduction of the strain energy of the system [30].

A significant fraction of dislocation density is used to maintain geometric compatibility at subgrain boundaries. Therefore, these dislocations may not be available for hardening, which explains the decrease in strain hardening rate with increasing polygonization. The material inside the cell walls tends to soften as dislocation walls form, with strain accumulating at the dislocation wall/cell interfaces. This heterogeneous distribution of dislocation density develops under conditions of multiple slip or primary slip plus strong secondary-slip activity [31]. The cell walls are also sometimes referred to as subgrain boundaries, and their depth appears to increase with the applied load, consequently increasing the affected volume with the load increase. When a sufficiently high stress or strain is reached, massive subsurface failure is instigated at the dislocation cell wall boundaries.

**Figure 15:**
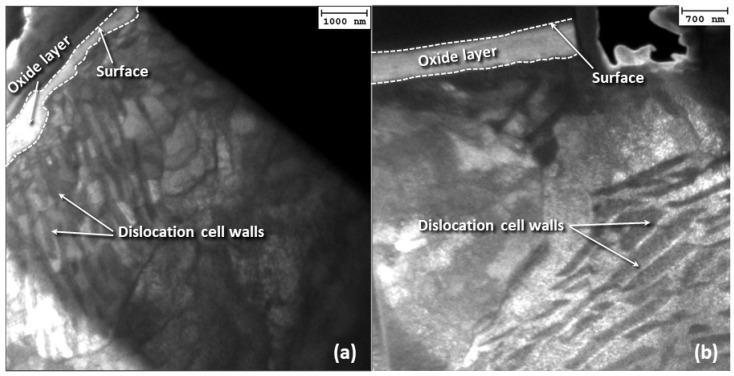
Dark-field TEM images showing the rotation of persistent slip bands in the subsurface of A216 carbon steel disk during fretting wear for 2 N load and sliding time equal to (**a**) 12 h and (**b**) 69 h.

Dislocation cell walls formed at varying depths, depending on applied load (Figure 14d–f). While the depth of dislocation cell walls increased with the load, catastrophic subsurface failure in a larger volume of material depended on the slip regime and number of sliding cycles. Although a change in the angle of dislocation cell wall formation and a significant increase in dislocation density at the dislocation cell walls did not occur with increasing load, a denser packing of dislocations in the cell walls did occur. Nevertheless, a rotation of the dislocation cells commenced with time (Figure 15), which most likely affected the fretting wear process by altering the oxidation rate, subsurface crack growth rate, and activation of additional slip systems. Specifically, if the dislocation cell walls were in fact areas that promoted an increased oxidation rate, a dislocation cell wall perpendicular to the surface would have caused a localized increase in oxidational rate at various locations of the oxide scale, causing some areas to oxidize faster than others. As these dislocation-rich walls rotated parallel to the surface, paths of enhanced oxygen diffusion were no longer oriented perpendicular to the surface, resulting in a more uniform oxide thickness.

Crack nucleation could initiate when the slip planes of persistent slip bands closely align with the maximum shear stress direction. However, as the size of dislocation cell walls and accumulated plastic strain increased with the progression of sliding, a lower stress was needed to initiate crack propagation along the interfaces of dislocation cell walls, and when the dislocation cell walls rotated parallel to the surface, fatigue cracks could still propagate. In delamination wear, subsurface cracks propagate parallel to the surface for considerable distances before they shear toward the surface, producing sheet-like particles [32]. Fatigue cracks may be affected by the rotation of highly strained cell boundaries parallel to the sliding direction, especially if cracks initiate in the shear direction and then continue to propagate under the effect of the tensile stresses arising at the trailing edge of asperity microcontacts. The fatigue cracks may then propagate more rapidly with the accrual of sliding cycles. Moreover, because the rotation of dislocation cells might also enhance the activation of various slip systems, many more slip systems aligned with the maximum shear stress as the cells rotated.

The progression of wear with the growth of dislocation cells/persistent slip bands can lead to a multitude of failures, depending on the growth characteristics of the cells and the stress distribution/wear conditions. If fretting wear is a balance between oxidational wear and subsurface failure at the dislocation cell walls, a massive failure could be instigated if the oxidational wear rate is less than the growth rate of dislocation cell walls at which a maximum density of dislocations has been attained. Since dislocations continue to proliferate as a result of cumulative plastic deformation, dislocation-free domains may eventually be consumed by dislocation-dense cell walls, increasing the proclivity for substantial subsurface failure. As long as the dislocation cell walls remained at an angle of 45°, crack propagation might have dominated the wear behavior. However, when the dislocation cell walls rotated to an angle parallel to the surface and oxidation rate increased due to the formation of other oxides, oxidational wear appeared to be the dominant process. Nevertheless, subsurface failure by crack propagation could still occur as soon as critical stresses developed at the cell wall boundaries.

### 4.2. Microstructure Evolution

An intriguing microstructural development was observed with the evolution of fretting wear (Figure 12, Figure 14a–c and Figure 15). The microstructures observed in the early (transient) stage of fretting wear (i.e., <12 h sliding time) were consistent with that of hypoeutectoid steel consisting of proeutectoid ferrite and pearlite at ambient temperatures under equilibrium conditions. However, the steady-state microstructure (i.e., ≥12 h sliding time) did not contain a pearlitic phase, but a uniform phase in the cell interiors. This trend can be explained as follows. Pearlite is a lath structure of eutectoid ferrite and cementite with numerous phase boundaries that can play a role as dislocation sources. As the dislocation cell walls formed, the dislocations from the cell interiors, including those associated with the pearlite microstructure, were driven toward the lower energy configuration of the cell wall, effectively dissociating the pearlitic microstructure. The carbon from the cementite was either redistributed into the cell interiors, supersaturating the ferrite with carbon, or followed the dislocations to the dislocation cell walls. Dissolution of the pearlitic microstructure can have many implications. Carbon redistribution into the cell interiors enhances the ferrite hardness. However, since ferrite has a low carbon solubility limit, it is energetically more favorable for carbon to diffuse along dislocation cores to the dislocation cell walls, leaving behind a malleable ferrite cell interior. Since finer ferrite grains improve strength and toughness in ferrite–pearlite microstructures, the dissolution of pearlite into dislocation cell structures may lead to the formation of an effectively larger grain (the cell interior), consequently decreasing the strength and toughness of the steel. Since ferrite is the softest phase in steel, the cells could be easily distorted with further wear. This would explain the fact that the dislocation cell walls appeared to have rotated to a direction parallel to the surface with time.

Regarding the stainless-steel microstructure of the pin sample, it was only damaged up to a depth of 500–700 nm (Figure 13). The absence of any dislocation structures suggests a beneficial alloying effect on the material’s resistance against fretting fatigue. Because steel alloying is accompanied by significant solid solution strengthening, dislocation movement was impeded, and the formation of dislocation structures was less favorable.

### 4.3. Oxidative Wear

The formation of a stable oxide scale can lessen the affinity of the surfaces for each other. A change in the composition of the interacting surfaces and associated change in solid solubility between the sliding surfaces can lower the interfacial adhesion, reducing the contribution of adhesive wear to the fretting wear process and causing other wear mechanisms, such as abrasive wear, oxidational wear, and subsurface fatigue, to dominate. Surface wear characterization (Figure 5, Figure 6, Figure 7, Figure 8 and Figure 9) revealed that the prevalent wear mechanisms varied from adhesive, abrasive, and fatigue wear of the oxide scale and the subsurface during the transient stage of fretting wear to mainly abrasive wear of the oxide scale and material transfer (adhesive wear) at steady state. Abrasive wear of the oxide scale would have remained dominant if the oxidation rate exceeded the abrasive wear rate and no other wear mechanism developed with accumulating oscillation cycles.

**Figure 16:**
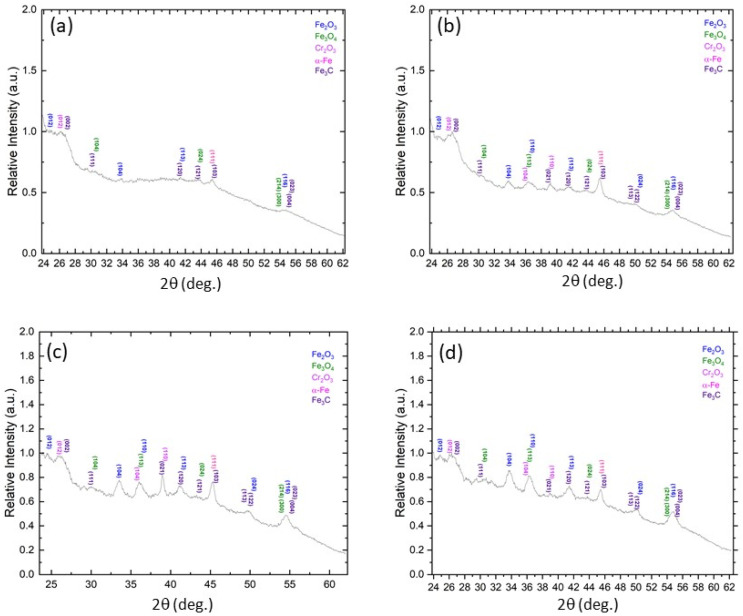
XRD spectra of wear debris collected from wear tests performed for 10 N load and sliding time: (**a**) 1 h, (**b**) 3 h (A significant increase of the relative intensity of the α-Fe and Fe_3_C peaks.), (**c**) 12 h (The relative peak intensity of the Cr_2_O_3_ and α-Fe and Fe_3_C peaks increased significantly.), and (**d**) 69 h (Although the relative intensity of the oxide peaks became mostly dominant, the α-Fe and Fe_3_C peaks are still present).

Oxide formation is a complex process described by many theories. It is generally accepted that iron and many of its alloys oxidize in a layered structure of varying stoichiometry. Closest to the iron substrate is wustite (FeO), followed by magnetite (Fe_3_O_4_), then farthest away, hematite (Fe_2_O_3_). The growth of FeO and Fe_3_O_4_ is controlled by the outward diffusion of cations, whereas the growth of Fe_2_O_3_ at the Fe_3_O_4_/Fe_2_O_3_ interface is dominated by the inward diffusion of anions [33,34]. More recently, others have suggested that Fe_2_O_3_ grows by outward cation migration [35]. Cation and anion migration is accelerated at higher temperatures; however, anything that can increase the diffusion rates of these ions enhances oxide growth. There is considerable controversy as to what temperatures are actually reached in the wear process. It is difficult to experimentally determine exact temperatures at the sliding interface, though some have attempted to measure flash temperatures by infrared technology [36,37,38]. It is widely believed that “hot spots” arise at asperity microcontacts established at the sliding interface. FeO is thermodynamically stable above 570 °C, below which it decomposes into Fe_3_O_4_ and α-Fe. Since XRD of wear particles did not reveal the formation of significant quantities of FeO in the present experiments (Figure 16), it may be inferred that the temperatures reached in the fretting experiments were likely below 570 °C.

During plastic deformation, dislocations nucleate and propagate to accommodate strain. It has been argued that oxidation is a stress/strain-aided process and that the development of stresses in the oxide as it thickens can halt further growth [6]. Dislocation density, oxide thickness, and lattice misfit between the oxide and the substrate affect stress-driven oxidation. Grain boundaries and dislocation cell walls are areas where diffusion is promoted and higher strain energy may evolve, increasing possible strain-driven diffusional processes. The TEM results of this study indicate an enhancement of the uniformity of the oxide scale with sliding time, whereas XRD of the wear particles shows a mixture of Fe_3_O_4_ and Fe_2_O_3_ debris (Figure 16). This change to a uniform oxidation suggests some alteration in primary oxide formed, modifying the Pilling–Bedworth ratio and thermal coefficient of expansion (which could impact the oxide–substrate interfacial adhesion) with increasing fretting time, subsequently leading to a less stressed oxide–substrate interface and the ability to retain a cohesive oxide layer on the surface. This difference in oxide thickness is evident in the TEM images. Specifically, while the oxide was unevenly distributed on the surface in the transient stage of sliding, at steady state, the oxide achieved uniformity unseen during previous time frames. As the dislocation cell structure became more pronounced and the dislocations polygonized to form cell walls, the dislocation cores could have served as pathways for oxygen transport into the substrate, promoting the formation of Fe_3_O_4_. Moreover, a change in the oxide debris color from reddish to blackish occurred with increasing sliding time, suggesting a possible change in oxide formation at the sliding surface with the progression of fretting wear. Fe_2_O_3_ is a reddish oxide, whereas Fe_3_O_4_ is a black oxide. All features appear to indicate a change in oxide formation with increasing sliding time. This can be explained by considering oxide formation in the context of ion migration. Any form of disruption to the matrix where ions travel, such as introduction of dislocations or formation of dislocation clusters, can change the rate of oxide formation. The development of dislocations during the wear process could trap iron cations, preventing ions from traveling toward the surface, in turn, decreasing the volume fraction of oxides forming at the surface. However, with the formation of channels, such as those made available by dislocation cell walls, an increase in oxygen anion transport was possible, increasing the amount of Fe_3_O_4_ formed at the surface of the steel sample. The rotation of these cell walls with increasing sliding time should have influenced oxygen diffusion into the subsurface, facilitating an increased oxidation rate parallel to the oxide surface rather than perpendicular to it. Consequently, a shift in major oxide formation at the surface may have occurred with increasing time and/or load, affecting the fretting wear process. In the present experiments, the oxide scale showed a considerably more even surface coverage that commenced with time, and did not appear to be completely removed at any location of the wear track.

Another important issue is the effect of mechanical properties of various iron oxides on the durability of the forming oxide scale. Since the hardness of Fe_3_O_4_ is lower than that of Fe_2_O_3_, any change in oxide scale composition from Fe_2_O_3_ to Fe_3_O_4_ with the progression of fretting wear would be accompanied by a decrease in hardness, which could significantly degrade the wear resistance of the steel surface. It is known that mechanically stable films of Fe_3_O_4_ form by a balanced diffusion of anions and cations into the film, making the bulk of the oxide more stable. Alternatively, Fe_2_O_3_ films are less stable because the Pilling–Bedworth ratio is high (~2.14), signifying a large internal stress, which, coupled with the expansion of the lattice due to oxide growth by anion migration, leads to the diminishment of the metal/oxide adhesion and, consequently, the growth of a thinner oxide scale [39]. This would explain why the oxide remained uniform over the entire wear track during steady-state fretting wear, but was uneven and discontinuously removed during the transient stage of fretting wear. A shift to a softer oxide could also lead to a faster oxidational wear rate and less spallation due to the decrease in the Pilling–Bedworth ratio. In any case, as long as the rate of oxide formation surpassed the rate of oxide removal, an even oxide coating could be anticipated.

Even though the surface wear characteristics displayed the operation of various wear mechanisms (Figure 5, Figure 6, Figure 7, Figure 8 and Figure 9), significant microstructural changes evolved in the subsurface and intensified with the increase of the load and the accumulation of sliding cycles (Figure 10, Figure 11, Figure 12, Figure 13, Figure 14 and Figure 15). The dislocation cells that formed in the subsurface could have been subjected to a massive failure when the cell walls reached critical strain levels for crack nucleation. This would suggest that subsurface fatigue could become the prominent failure mechanism under appropriate conditions, such as longer fretting time, in conjunction with the dominance of abrasive wear of the oxide scale at the sliding interface. The relative intensity of the Fe_3_C and α-Fe peaks in the XRD data appeared to increase with increasing sliding time from 1 to 12 h (Figure 16a–d), implying there could be an increase in substrate material contained in the wear debris with increasing sliding time. After sliding for 69 h, there were still notable amounts of α-Fe and Fe_3_C in the wear debris (Figure 16d).

### 4.4. Material Transfer

In addition to the formation of wear debris, continuous back-and-forth transfer of worn material and temporary attachment to the rubbing surfaces is a commonly encountered process, especially under dry sliding conditions and with material systems exhibiting a high degree of metallurgical compatibility [40,41]. The much higher Cr content of 410 stainless steel than that of A216 carbon steel served as an excellent marker for determining transfer of the pin material to the disk surface during fretting wear (Figure 5, Figure 6, Figure 7, Figure 8 and Figure 9). The background level of Cr seen outside the wear scars in all of the carbon steel maps is due to the small amount of Cr in this steel. Material transfer from the pin surface to the disk surface during the transient stage of fretting wear increased with the load (Figure 5). Little to no material transfer was found for 2 N load (slip). Increasing the load to 10 N (slip) and 15 N (stick-slip) led to significant material transfer at the center of the wear scar, attributed to the much higher surface shear traction developed under the high-load, stick-slip contact conditions. Significant material transfer, characterized by an uneven Cr distribution, commenced in the transient stage of fretting wear (Figure 5, Figure 6, Figure 7 and Figure 8), as opposed to fairly evenly distributed Cr and less material transfer under steady-state fretting wear (Figure 9). This can be explained by considering that the contact pressure was unevenly distributed during the transient stage of fretting wear due to the poor conformability of the surfaces and the formation of rough adhesion and abrasion wear marks, which produced local high-pressure spikes (and shear tractions) conducive to material transfer. Moreover, the XRD data of the wear debris on the carbon steel disk surfaces suggest that chromium oxide (Cr_2_O_3_) was also present (Figure 16).

## 5. Conclusions

The microstructural evolution and dominant wear mechanisms of carbon steel sliding against stainless steel under small-amplitude oscillatory sliding (fretting) conditions were investigated experimentally. A synergy between oxidational wear and subsurface fatigue characterized the fretting wear process. At light loads (slip), oxidational wear prevailed, as subsurface plasticity was not so severe as to cause failure. Nevertheless, the dislocation structure that formed in the subsurface affected the growth of the oxide scale, causing a change in oxide stoichiometry over time. At high loads (stick-slip), the accumulation and alignment of dislocation cell walls led to material failure by delamination. The depth of sub-cell boundaries increased with the load, with dislocation cells developing under both slip and stick-slip conditions. The dislocation cells occupied a larger fraction of the subsurface after the transition from slip to stick-slip conditions, and the depth of the damaged layers was affected by the depth distribution of the shear stress.

Furthermore, the depth of dislocation cells increased over time under slip contact conditions, indicating a slower wear rate than the formation rate of dislocation cells and that long-term catastrophic failure could result from cell wall-induced subsurface delamination. With the accumulation of oscillation cycles, the dislocation cells rotated from being aligned with the maximum shear stress to being nearly parallel to the surface. This rotation of the dislocation cells affected crack nucleation and growth as well as oxide scale formation at the sliding surface, suggesting direct implications for the service lifetime of engineering components experiencing oscillatory contact.

A transition from non-uniform to uniform oxide scale formation during steady-state fretting wear occurred under light-load (slip) conditions. This was accompanied by a change in oxide stoichiometry from Fe_2_O_3_ to Fe_3_O_4_ with increasing sliding time, leading to the formation of a uniform oxide scale, attributed to the formation of dislocation cells in the subsurface. The dislocation-rich cell walls served as enhanced diffusion sites transporting oxygen ions into the steel and augmenting the growth of Fe_3_O_4_ at the surface. Despite the lower hardness of Fe_3_O_4_ than that of Fe_2_O_3_, the smaller Pilling–Bedworth ratio of Fe_3_O_4_ yielded lower stresses in the oxide scale and better adhesion to the steel substrate. An even oxide scale formed when the growth rate exceeded the wear rate of the oxide scale.

The SEM and EDS results revealed that the surface wear mechanisms varied from adhesive, abrasive, and subsurface fatigue during the transient stage of fretting wear to mainly abrasive wear of the oxide scale and some material transfer due to adhesion at steady state. Stress-induced microstructural evolution was observed with the progression of fretting wear. Dislocation cells developing in the subsurface with the accumulation of fretting cycles experienced massive failure at critical strain levels, resulting in crack nucleation and subsequent crack growth in the subsurface for substantial time before shearing toward the sliding interface to produce wear particles at steady state.

The findings of this investigation provide new insight into the highly chemomechanical nature of fretting wear and highlight the importance of combining surface with subsurface analytical techniques to unravel key characteristics illustrative of this poorly understood wear process.

## Figures and Tables

**Table 1:** Composition of disk and pin specimens.

Element	Steel Composition (wt%)	
	A216 Carbon Steel	410 Stainless Steel
Carbon	0.25–0.3	<0.15
Chromium	0.5	11.5–13.5
Manganese	0.7–1.2	<1.0
Phosphorous	0.035	<0.04
Sulfur	0.035	<0.03
Silicon	0.6	<1.0

## Data Availability

All data generated or analyzed during this study are included in this published article.
